# Contextual differences considered in the Tunisian ADOLOPMENT of the European guidelines on breast cancer screening

**DOI:** 10.1186/s12961-021-00731-z

**Published:** 2021-05-13

**Authors:** Lara A. Kahale, Hella Ouertatani, Asma Ben Brahem, Hela Grati, Mohammed Ben Hamouda, Zuleika Saz-Parkinson, Elie A. Akl

**Affiliations:** 1 American University of Beirut, Beirut, Lebanon; 2 L’Instance Nationale de l’Evaluation et de l’Accréditation en Santé (INEAS), Tunis, Tunisia; 3 European Commission, Joint Research Centre, Ispra, Italy; 4 McMaster University, Hamilton, Canada

**Keywords:** Practice guideline, Adaptation, GRADE, Evidence-based medicine, Tunisia, Breast cancer, European Commission Initiative, ADOLOPMENT

## Abstract

**Background:**

Breast cancer is a common disease in Tunisia and is associated with high mortality rates. The “Instance Nationale de l’Evaluation et de l’Accréditation en Santé” (INEAS) and the Tunisian Society of Oncology decided to develop practice guidelines on the subject. While the development of de novo guidelines on breast cancer screening is a demanding process, guideline adaptation appears more appropriate and context sensitive. The objective of this paper is to describe the adaptation process of the European Guidelines on Breast Cancer Screening and Diagnosis to the Tunisian setting in terms of the methodological process, contextual differences between the source and adoloped guideline, and changes in the recommendations.

**Methods:**

We used the ‘Grading of Recommendations Assessment, Development and Evaluation’ (GRADE)-ADOLOPMENT methodology to prioritize the topic, select the source guideline, and prioritize the questions and the outcomes. Once the source guideline was selected—the European Breast Cancer Guidelines—the European Commission´s Joint Research Centre shared with the project team in Tunisia all relevant documents and files. In parallel, the project team searched for local studies on the disease prevalence, associated outcomes’ baseline risks, patients’ values and preferences, cost, cost-effectiveness, acceptability, and feasibility. Then, the adoloping panel reviewed the GRADE evidence tables and the Evidence to Decision tables and discussed whether their own judgments were consistent with those from the source guideline or not. They based their judgments on the evidence on health effects, the contextual evidence, and their own experiences.

**Results:**

The most relevant contextual differences between the source and adoloped guidelines were related to the perspective, scope, prioritized questions, rating of outcome importance, baseline risks, and indirectness of the evidence. The ADOLOPMENT process resulted in keeping 5 out of 6 recommendations unmodified. One recommendation addressing “screening versus no screening with ultrasound in women with high breast density on mammography screening” was modified from ‘conditional against’ to ‘conditional for either’ due to more favorable ratings by the adoloping panel in terms of equity and feasibility.

**Conclusion:**

This process illustrates both the feasibility of GRADE-ADOLOPMENT approach and the importance of consideration of contextual evidence. It also highlights the value of collaboration with the organization that developed the source guideline.

## Background

Breast cancer represents the second most prevalent cancer in the world affecting 2.1 million women each year [1]. According to the latest ‘Global Cancer Incidence, Mortality and Prevalence’ (GLOBOCAN) estimates, the incidence has increased by more than 20% and mortality by 14% in 4 years. The incidence rates are higher in the most developed countries, but mortality rates remain much higher in low-income countries, reflecting a gap in the early detection and access to treatment. In Tunisia, it represents the most common type of cancer; among 100,000 women, there are 32.2 incident cases and 10.3 related deaths each year [1].

To address this public health problem, the “Instance Nationale de l’Evaluation et de l’Accréditation en Santé” (INEAS) and the Tunisian Society of Oncology decided to develop practice guidelines on the subject. Indeed, guidelines can enhance evidence-based practice and reduce variability in practice [2].

However, developing guideline de novo (i.e., ‘from scratch’) can be a demanding process in terms of time, human, and financial resources. Alternative options to de novo development include adopting or adapting guidelines developed by others [3, 4]. While adoption of a guideline can be done quickly and with fewer resources, it might be inappropriate when contextual differences between the original and target setting exist. In these cases, adaptation of guidelines is a more appropriate approach as it takes into account contextual differences [4].

A methodological survey identified eight methodologies for the adaptation of health guidelines [4]. The ‘Grading of Recommendations Assessment, Development and Evaluation’ (GRADE)-ADOLOPMENT, one of these methodologies, combines the advantages of adoption, adaptation and de novo guideline development which allows the creation of recommendations appropriate to the context [3]. GRADE-ADOLOPMENT is based on three cornerstones: (1) identifying and prioritizing credible existing relevant guidelines or evidence syntheses (2) evaluating and completing the GRADE Evidence to Decision (EtD) frameworks for each of the recommendations; and (3) deciding on a final adoption, adaptation or de novo development for each of the recommendations [5].

The objective of this paper is to describe the project in terms of the methodological process, contextual differences between the source and adoloped guideline, and changes in the recommendations.

## Methods

### Overall process

The process of this project is based on the steps of the Guidelines 2.0 checklist [6], and the GRADE-ADOLOPMENT approach [3]. We used the GRADEpro- ‘guideline development tool (GDT) software [7] to develop GRADE evidence tables and EtD frameworks [5]. The GRADE evidence table provides the effect estimates for each outcome of interest and the associated certainty of evidence [8].

**Fig. 1:**
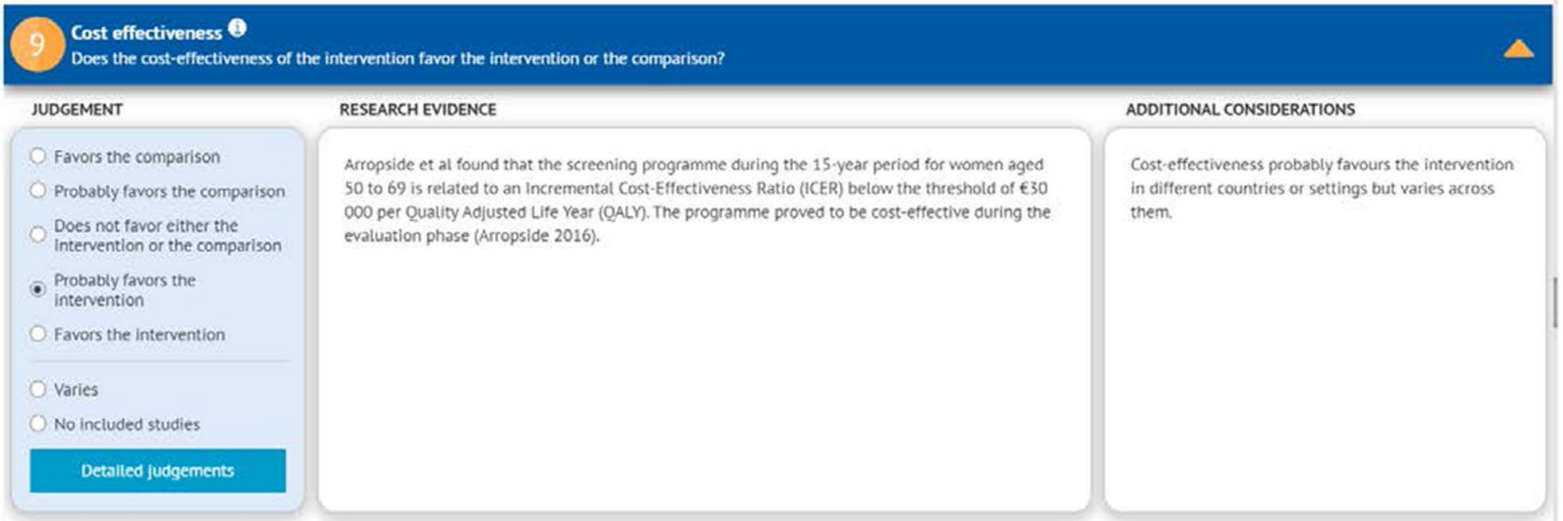
Example for the cost-effectiveness section from an EtD framework

The EtD table includes information on the following criteria: desirable and undesirable effects, certainty of evidence, certainty about or variability in values and preferences, cost, and cost-effectiveness, equity, feasibility, and acceptability [9–11]. The information included for each EtD criterion consists of judgment, research evidence, and additional considerations. Figure 1 shows this information displayed in columns for one of the factors (cost effectiveness used as an illustrative example) [12].

We describe below the methodological aspects of the project most relevant to the ADOLOPMENT process.

### Contributors

INEAS is an independent public authority that contributes to the regulation of the health system in Tunisia through quality and efficiency. The guideline project was a collaborative effort between INEAS and the Tunisian Society of Oncology. The Deutsche Gesellschaft für Internationale Zusammenarbeit (GIZ) GmbH funded the study, while the ‘American University of Beirut’ (AUB) GRADE (Grading of Recommendations Assessment, Development and Evaluation) center provided the methodological support.

Two major groups were involved: the project team and the guideline panel. The project team consisted of four members from INEAS (ABB, HO, HG, MH) and two members of the AUB GRADE center (LK, EA). The guideline panel consisted of 12 local experts including medical and surgical oncologists, gynecologists, family medicine, radiologists, guideline methodologists, and governmental representatives. None of the panelists had financial conflict of interest.

### Prioritization of the topic

The project team initially considered the following four topics: breast cancer screening, colorectal cancer screening, hypertension, and management of pain. The team then conducted a priority setting exercise to prioritize one of those topics [13, 14]. The factors considered for priority setting included: public health burden; avoidable mortality and morbidity; economic burden on the health care system and patient; emerging diseases or emerging care options; potential impact of intervention on health outcomes, economy, health care system, and equity; variation in clinical practice; and rapidly changing evidence [13, 14]. Eventually, the project team prioritized the topic of breast cancer screening as it was rated the highest.

### Selection of the source guideline

The project team systematically searched for existing guidelines on breast cancer screening published after 2016, to ensure they were up to date. The group searched MEDLINE, GuidelineCentral, Guideline International Network database, and websites of guideline producing agencies such as the National Institute for Health and Care Excellence, National Comprehensive Cancer Network, Scottish Intercollegiate Guidelines Network, World Health Organization (WHO), Belgian Health Care Knowledge Center, and Agency for Healthcare Research and Quality.

**Fig. 2:**
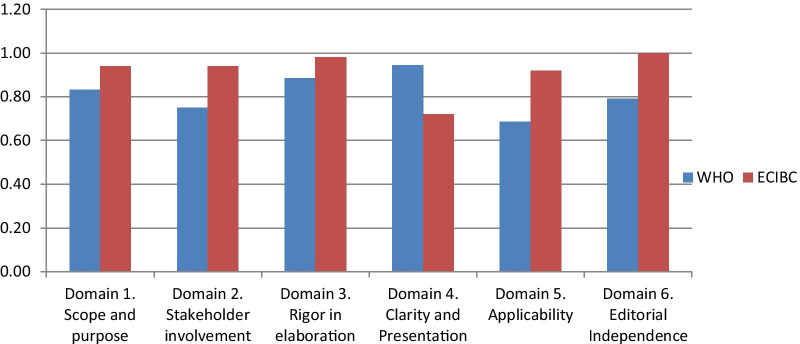
Scoring of the two potentially eligible guidelines as per the AGREE II tool. *WHO* World Health Organization, *ECIBC* European Commission Initiative on Breast Cancer

The search identified 124 unique citations. The title and abstract screening yielded seven citations as potentially eligible (on breast cancer). The full-text screening of the seven citations identified two relevant guidelines that were based on systematic reviews and developed using the GRADE approach [15, 16]. Two members from INEAS (HO, ABB) independently assessed the methodological rigor and transparency of each of the two guidelines using the AGREE II tool [17]. The project team selected the breast cancer screening guidelines developed by the European Commission Initiative on Breast cancer (ECIBC) as it scored the highest on the ‘Appraisal of Guidelines for Research and Evaluation’ (AGREE) II tool as shown in Fig. 2. [18].

### Prioritization of questions and outcomes

For prioritizing the questions and the outcomes, the project team organized a face-to-face panel meeting in September 2018. The panelists anonymously rated the importance of each of the nine screening questions addressed in the source guideline at the time this ADOLOPMENT process was started. They used a scale of 1–9 (least important—most important) and considered the relevance of the populations and interventions addressed by each question. Then, the panel discussed the rating results and selected the final set of questions through consensus.

Similarly, the panel rated the importance of the outcomes defined in each of the questions of the source guideline on a scale of 1–9 (7–9 indicates outcome is critical for decision-making, 4–6 indicates it is important, and 1–3 indicates it is not important for decision-making) [6].

### Gathering the evidence and preparing the EtD

For the evidence on health effects, the ECIBC team (ZSP) shared with the project team the evidence syntheses reports and other relevant documents from the source guideline, including the GRADEpro files for GRADE evidence tables and EtD frameworks. Updating the search for the health effects evidence was not required to identify new evidence given the short timeline between the publication of the source guidelines and the ADOLOPMENT process by the Tunisian panel.

**Fig. 3:**
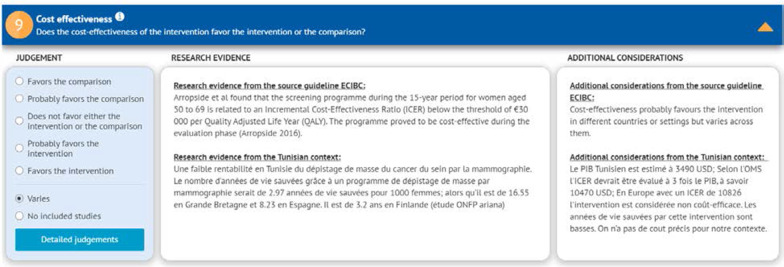
Display of the standard EtD table adapted for use in the Tunisian ADOLOPMENT project

For the contextual evidence, the project team searched for local studies on the disease prevalence, associated outcomes’ baseline risks, patients’ values and preferences, cost, cost-effectiveness, acceptability, and feasibility. As the team did not identify much of the needed data from published studies, it solicited them from panel members and searched for studies from contexts similar to that of Tunisia (i.e., Arabic countries of North African countries). The local baseline risk was integrated in the evidence summary tables. Then we adapted the standard EtD table in GRADEpro to use in the ADOLOPMENT process, as the GRADEpro-GDT ADOLOPMENT module had not been developed at that point. For each criterion we reproduced the ‘research evidence’, the ‘additional considerations’, and the ‘judgments’ from the source guideline. In addition, we added under ‘research evidence’ any identified local data (Fig. 3).

### Finalizing the recommendations

In December 2018, the panel reviewed the GRADE evidence tables and the EtD frameworks. For each healthcare question, and for each EtD criterion, the panel started by reviewing the ‘research evidence’, the ‘additional considerations’, and the ‘judgments’ from the source guideline, as well as any identified ‘research evidence’ from the Tunisian setting. Next, they added their own ‘additional considerations’ for each criterion and discussed whether they would modify the judgments from the source guideline (Fig. 3). After going through all the criteria, they considered how the corresponding judgments were modified, and accordingly decided whether to modify the source recommendation and the accompanying remarks.

## Results

### Contextual differences between source and adoloped guidelines

We present in Table 1 below the most relevant contextual differences between the source and ADOLOPMENT guidelines: perspective, scope, prioritized questions, rating of outcome importance, baseline risks, and indirectness of the evidence.

**Table 1:** Contextual differences between the source (ECIBC) and adoloped (Tunisian) guidelines

Guideline Item	ECIBC Guideline	Tunisian Guideline	Rationale for change by Tunisian panel
Perspective	Population	Individual	Organized mammography screening programs not available in Tunisia
Scope	Screening and diagnosis	Screening	Feasibility considerations; first ADOLOPMENT experience
Prioritized questions	9*	6	3 questions on screening using tomosynthesis were dropped, as tomosynthesis is not used in Tunisia as a screening tool
Rating of outcome importance	‘All-cause mortality’ important;‘overdiagnosis’ critical	‘All-cause mortality’ not important;‘overdiagnosis’ important	Change in perspective (from population to individual)
Baseline risks	Breast cancer incidence and breast cancer mortality rate from the meta-analysis control arm data	Lower breast cancer incidence, higher breast cancer mortality rate	Overall, the baseline risk of breast cancer mortality in Tunisia was assumed to be similar to that in Europe
Indirectness of the evidence	Judgment of indirectness made in the source guideline	Judgment of no (further) rating down of certainty of evidence for indirectness	Based on the consideration of how the characteristics of the populations or the interventions in the Tunisian setting compare to the setting of the source guideline

*By the time the ADOLOPMENT process was initiated, the source guideline had published recommendations for nine questions only

### Changes in the recommendations

**Fig. 4:**
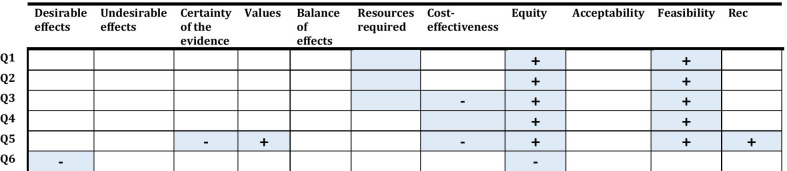
Changes made by the adoloping panel to the judgments made by the source guideline panel for the different EtD criteria and the recommendation statements, for each of the six questions. Rec: Recommendation. The blue shade refers to the changes made by the adoloping panel to the judgments made by the source guideline panel for the different EtD criterion and the recommendation statements, for each of the six questions. +  refers to the change in judgment that made the corresponding factor more favorable. − refers to the change in judgment that made the corresponding factor less favorable

Figure 4 indicates whether the judgments made by the source panel were modified by the adoloping panel for the different EtD criterion and the recommendation statements, for each of the six questions. The ADOLOPMENT process resulted in keeping 5 out of 6 recommendations unmodified. The modified recommendation addressed “screening versus no screening with ultrasound in women with high breast density on mammography screening”. The panel modified the recommendation from ‘conditional against’ to ‘conditional for either’ due to more favorable ratings by the adoloping panel in terms of equity and feasibility. For each of the five remaining unmodified recommendations, the adoloping panel had different judgments (relative to the source guideline) for at least one of the EtD criteria (range 2–4 criteria).

## Discussion

### Summary

The ADOLOPMENT of the European Guidelines on Breast Cancer Screening and Diagnosis to the Tunisian setting illustrates the feasibility of carrying out this process with limited resources and in a short period of time (3 months). We have highlighted the complete methodological process followed which led to six contextual differences between the source guideline and the Tunisian one, and changes in the recommendations.

### Facilitators and implementation considerations

A major facilitator to this ADOLOPMENT project was the collaboration between the two teams of the source and ADOLOPMENT guidelines. The ECIBC guideline project team allowed the unrestricted use of their recently published guideline and related material as the basis for the ADOLOPMENT process [18]. Another major facilitator is the fact that the two guideline efforts used the same methodology (i.e., GRADE), and the same tools (e.g., RevMan, GRADEPro-GDT). On the other hand, one major challenge was the lack of published local evidence from Tunisia for values and preferences, and economic implications. The judgments made for those criteria relied mainly on expert evidence provided by the panelists [19].

A clear advantage of guideline adaptation is the ability to present the adoloping panel with evidence that has already been synthesized for the source guideline. However, it is not clear whether the panel should be also presented with the EtD sections completed by the source guideline’ panel. These sections include ‘judgments’ and ‘additional considerations’ made for the different EtD criteria, and the final ‘recommendation’. In principle, there are three possible approaches to sharing information from the source guideline with the adoloping panel, as illustrated in Table 2.

**Table 2:** Three possible approaches to sharing with the adoloping panel information from the source guideline

Approach	Contextual evidence	Information from the source guideline			
		For each EtD criterion			Recommendation
		Synthesized evidence	Judgment	Additional considerations	
A	x	x	x	x	x
B	x	x	x	x	
C	x	x			

Approach A would allow the panel to build on the source guideline’s full information and decide whether to modify any of the judgments or recommendation. Approach B would allow the panel to build on the source guideline’s information except for the recommendation. The panel would decide whether to modify any of the judgments but develop the recommendation independently. Approach C would allow the panel to make their judgments and develop the recommendations independently, taking into account only the evidence synthesized for the source guideline and the contextual evidence. This approach C would require more extensive discussions and time compared with the two other approaches. It could be used in scenarios when judgments and additional considerations are not available, when there are concerns about the judgments made by the source panel, and when preferred by the adoloping panel.

While we have used approach C in previous ADOLOPMENT projects [20–25], we opted to go for approach B in this project. The decision was driven by the preference of the adoloping panel, and by the scarcity of local evidence from Tunisia. These experiences and the principles for ADOLOPMENT [3] formed the basis for the development of the GRADEpro-GDT ADOLOPMENT module [7] which includes the following:

1. for each EtD criterion, the synthesized evidence on health effects, judgments, and additional considerations already made by the source guideline panel (reproduced from the source guideline);
2. for each EtD criterion, the contextual evidence, judgments, and additional considerations, to be made by the ADOLOPMENT guideline panel;
3. the recommendation developed by the source guideline panel (reproduced from the source guideline);
4. the recommendation to be developed by the adoloping panel.

However, including the elements from the source guideline (#1 and #3 above) are optional to allow the panelists select one of the three approaches discussed above (Table 2).

**Fig. 5:**
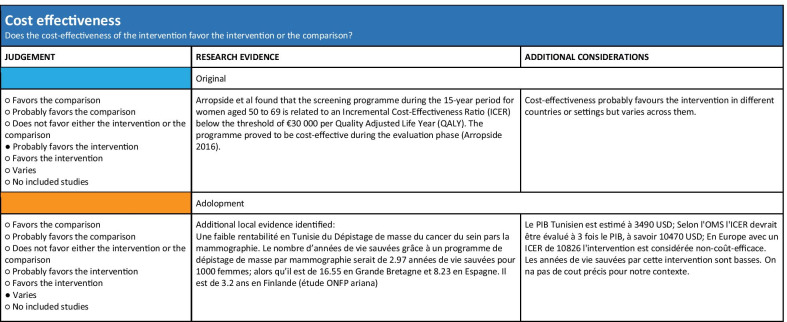
Display of how the EtD used in the Tunisian ADOLOPMENT would look using the GRADEpro-GDT ADOLOPMENT module

**Fig. 6:**
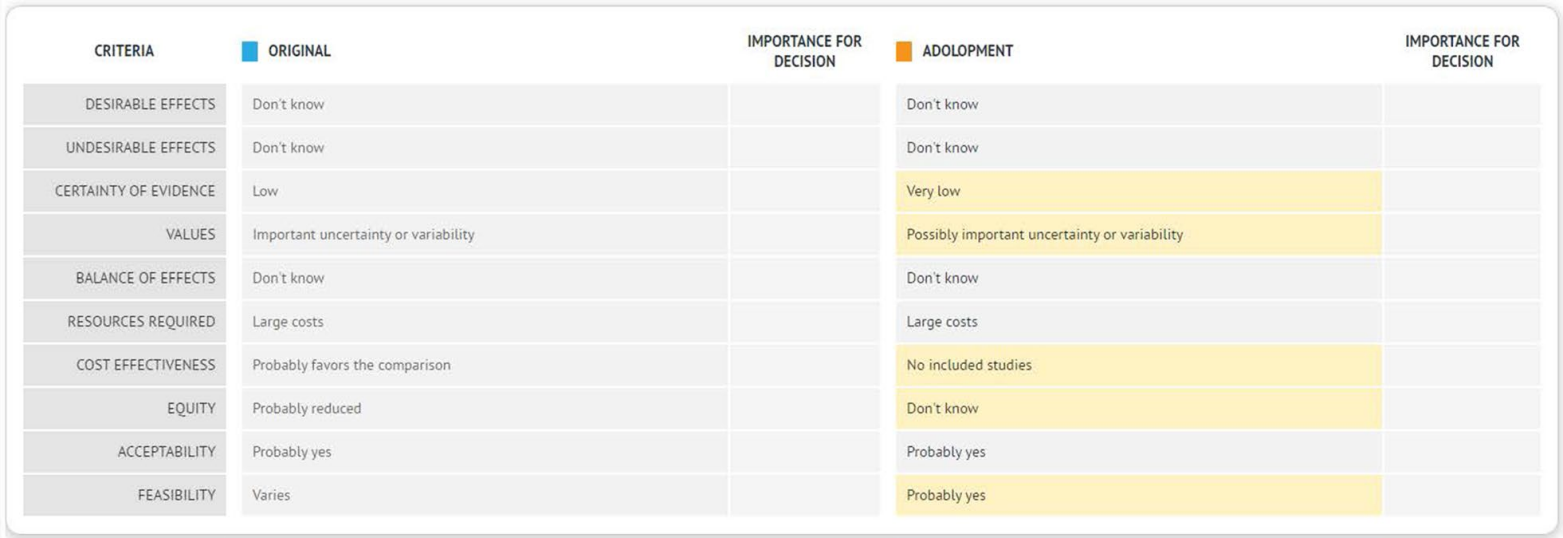
Display of how the summary of the judgments by both the source and the adoloped panels across all criteria in GRADEpro-GDT ADOLOPMENT module

The information from the source guideline is reproduced but not editable. For the adoloped guideline, a blank section allows the project team to add any local evidence, and the adoloping panelists to include their own judgments, their own additional considerations, and their own recommendation. Figure 5 shows how the EtD used in this project (Fig. 3) would look using the GRADEpro-GDT ADOLOPMENT module. Figure 6 shows how the summary of the judgments across all criteria by both the source and the adoloped panels would look using the GRADEpro-GDT ADOLOPMENT module.

### Implications for practice

This project illustrates a number of facilitators for guideline ADOLOPMENT, including (1) collaboration with the organization that developed the source guideline; (2) same methodology (GRADE) used for the source guideline development and the adoloped guideline; (3) availability of contextual evidence; (4) availability of an adaptation module in a guideline development tool (e.g., GRADEPro-GDT); and (5) engagement of panelists in the ADOLOPMENT process.

An organization aiming to facilitate the adaptation of its recommendations need to strategically optimize their ‘adaptability’. This can be achieved through using structured methodology, such as GRADE, making explicit detailed judgments related to the certainty of evidence and strength of recommendation. Such a methodology would also allow the presentation and judgment of the health effects and of the contextual factors separately, as the latter are more likely to be judged differently during the adaptation process. Providing open access to all relevant material (e.g., evidence syntheses, EtD tables) would also optimize adaptability of recommendations.

### Implications for research

As illustrated by Fig. 4, the adoloping panel might change several judgments for some EtD criteria, without leading to a change in the recommendation. It would be interesting to explore to what extent this observation applies to other guideline adaptation efforts. In addition, there is a need to evaluate the feasibility of the three approaches of sharing with the adoloping panel information from the source guideline, and their acceptability by the panelists and methodologists. Finally, it would be helpful to develop an extension to the G-I-N-McMaster checklist for guideline development [6], to support groups adapting guidelines. [26] Of similar importance is the development of an extension of the RIGHT statement to improve the reporting of adapted guidelines. [27, 28]

## Conclusion

This process illustrates both the feasibility of GRADE-ADOLOPMENT approach and the importance of consideration of contextual evidence. It also highlights the value of collaboration with the organization that developed the source guideline.

## Data Availability

Available upon request.

## Abbreviations

AGREE: Appraisal of Guidelines for Research and Evaluation
AUB: American University of Beirut
ECIBC: European Commission Initiative on Breast Cancer
EtD: Evidence to decision
GIZ: Deutsche Gesellschaft für Internationale Zusammenarbeit
GLOBOCAN: Global Cancer Incidence, Mortality and Prevalence
GRADE: Grading of Recommendations Assessment, Development and Evaluation
INEAS: Instance Nationale de l’Evaluation et de l’Accréditation en Santé
WHO: World Health Organization
